# How Angular Velocity Features and Different Gyroscope Noise Types Interact and Determine Orientation Estimation Accuracy

**DOI:** 10.3390/s150923983

**Published:** 2015-09-18

**Authors:** Ilaria Pasciuto, Gabriele Ligorio, Elena Bergamini, Giuseppe Vannozzi, Angelo Maria Sabatini, Aurelio Cappozzo

**Affiliations:** 1 Interuniversity Center of Bioengineering of the Human Neuromusculoskeletal System, Department of Movement, Human and Health Sciences, University of Rome “Foro Italico”, Piazza Lauro de Bosis 15, 00135 Roma, Italy; E-Mails: ilaria.pasciuto@uniroma4.it (I.P.); elena.bergamini@uniroma4.it (E.B.); aurelio.cappozzo@uniroma4.it (A.C.); 2 The BioRobotics Institute, Scuola Superiore Sant’Anna, Piazza Martiri della Libertà 33, 56124 Pisa, Italy; E-Mails: g.ligorio@sssup.it (G.L.); angelo.sabatini@sssup.it (A.M.S.)

**Keywords:** 3D orientation, MEMS gyroscopes, noise sources, motion analysis, gait, biomechanics, human, numerical integration, inertial sensors

## Abstract

In human movement analysis, 3D body segment orientation can be obtained through the numerical integration of gyroscope signals. These signals, however, are affected by errors that, for the case of micro-electro-mechanical systems, are mainly due to: constant bias, scale factor, white noise, and bias instability. The aim of this study is to assess how the orientation estimation accuracy is affected by each of these disturbances, and whether it is influenced by the angular velocity magnitude and 3D distribution across the gyroscope axes. Reference angular velocity signals, either constant or representative of human walking, were corrupted with each of the four noise types within a simulation framework. The magnitude of the angular velocity affected the error in the orientation estimation due to each noise type, except for the white noise. Additionally, the error caused by the constant bias was also influenced by the angular velocity 3D distribution. As the orientation error depends not only on the noise itself but also on the signal it is applied to, different sensor placements could enhance or mitigate the error due to each disturbance, and special attention must be paid in providing and interpreting measures of accuracy for orientation estimation algorithms.

## 1. Introduction

One of the main issues in human movement analysis is the accurate estimation of the three-dimensional (3D) orientation of a body segment, relative to a global Earth-fixed reference frame. In fact, body segment orientation is needed to estimate both kinematic and kinetic joint parameters in a variety of contexts: for instance, in clinical contexts, they allow to assess the functional outcome of treatments [1,2]; in sports, to evaluate the outcome of training protocols [3]; and in activity monitoring, to identify features such as walking instability or fall risk, which are of great concern in pathological and elderly populations [4,5].

Among the technologies currently available for the estimation of a rigid body 3D orientation in human movement analysis, Magneto-Inertial Measurement Units (MIMUs) are becoming increasingly popular. Recent advances in the field of Micro Electro-Mechanical Systems (MEMS), in fact, have allowed manufacturing sensors characterized by reduced dimensions, relatively low-cost, and easy set-up, which enable unconstrained motion monitoring [6,7]. MIMUs consist of a set of gyroscopes, accelerometers and magnetic sensors, which provide 3D information concerning the angular velocity, the linear accelerations the MIMU is subject to and the local magnetic field, respectively, expressed in a Cartesian coordinate system fixed to the MIMU itself.

In principle, the 3D orientation of the MIMU (and therefore of the body it is attached to) can be obtained through the gyroscope signal alone, by numerically integrating the non-linear kinematic equations that relate the angular velocities with the time-derivative of the orientation, regardless of the formalism employed for its description [8]. However, the integration process leads to errors that grow over time and, in addition, the initial conditions for the integration often need to be determined. In order both to reduce error propagation and to obtain the integration process initial conditions, a sensor fusion approach is often followed [9]. In such cases, the gyroscope signal still represents the basis for MIMU orientation estimation, but its information is refined with the data from the accelerometers and magnetic sensors.

Nevertheless, there are cases in which the sensor fusion approach has been proved not to represent a real added value with respect to the numerical integration alone [9]. This occurs, for example, when short durations (less than 20 s) are involved, or in case the characteristics of the analyzed movement do not allow for the accelerometer and magnetic sensor data to be effectively exploited to compensate the integration drift (persistence of non-zero inertial accelerations or presence of ferromagnetic disturbances). In such cases, the accuracy of the 3D body orientation is entirely determined by the quality of the gyroscope signal. Therefore, the effect of the types of noise that typically affect the gyroscope on the accuracy of the orientation estimation is particularly worthy of consideration, regardless of whether the orientation is estimated through direct integration or through the application of a sensor fusion technique.

MEMS gyroscope measurements are affected by errors, due to several types of noise, which may be classified as deterministic or stochastic [10]. Deterministic (or systematic) errors can be accounted for in the data processing if quantified correctly, whereas stochastic errors cannot be foreseen but can be statistically modelled. Four noise types can be considered as the most relevant [11]: constant bias, scale factor, white noise and bias instability. The first two are associated with deterministic errors and are hereinafter referred to as deterministic noise types, whereas the last two are associated to stochastic errors and are hereinafter referred to as stochastic noise types.

The constant bias is the angular velocity value provided by the gyroscope when it is not undergoing any rotational movement with respect to an Earth-fixed frame. In order to compensate for this offset, its value is usually estimated through a procedure called bias capture, which consists in averaging the gyroscope signals obtained during a static acquisition (e.g., placing the gyroscope on a fixed surface), when the expected angular velocity is null. Although most of the gyroscope offset can be accounted for with this procedure, the averaging process can entail uncertainties which lead to a residual bias that remains uncompensated. Its effect is that of a steadily growing angular error in time [10,12].

The scale factor is the ratio between the gyroscope-measured values and the actual angular velocity and is due to errors affecting the sensor’s calibration performed during its manufacturing. To assess how far the gyroscope scale factor is from the ideal conditions (unity), the sensor can either be rotated at a predefined angular velocity, which is then compared to the measured signal, or it can be rotated by a known angle, which is then compared to the integral of the kinematic equation relating the angular velocity with the time-derivative of the orientation [13]. The former procedure is more accurate but requires specific instrumentation (e.g., turntables), whereas the latter does not require additional instrumentation but provides less accurate estimations. The error in orientation produced by the scale factor is proportional to the scale factor itself and to the duration of the motion [12].

The white noise is a sequence of zero-mean uncorrelated random values, which fluctuate at a much greater rate than the sampling frequency of the gyroscope and are due to thermo-mechanical causes. Its integration leads to a zero-mean random walk process, with a standard deviation that grows with the square root of time [12].

Finally, the bias instability is a stochastic error that indicates how unstable is the bias of a gyroscope over a certain specified period of time (gyroscope drift rate) [11,14]. Several types of stochastic processes, including flicker noise and rate random walk, have been considered to describe and model the bias instability [14]. Irrespective of the actual origin of the gyroscope drift rate, one commonly used approach is to model it as a Gaussian random walk process [15]. In the present work, this approach was adopted and the standard deviation of the Gaussian random walk process was used to describe the bias instability.

Although the effect of the mentioned noise types on the estimated orientation over time has already been studied in the literature [12,16], to the authors’ knowledge no study has been published concerning whether and to what extent the angular velocity signal itself affects the orientation error due to the mentioned types of noise. Due to the non-linear relationship between angular velocity and orientation, in fact, it is unclear whether the magnitude of the angular velocity and its distribution across the three gyroscope axes may alter the effect of the considered noise types, and therefore whether there are experimental conditions which may mitigate or enhance the orientation error.

In light of the above considerations, the main objective of the present study is to assess the influence of the magnitude and 3D distribution of the angular velocity signal on the orientation error due to the four above-mentioned types of noise that affect MEMS gyroscopes, in reference to the human motion analysis context. To this aim, a simulation approach was followed to corrupt reference angular velocity signals, considered noise-free, with each considered noise type, and both the reference and corrupted signals were integrated to obtain the corresponding orientations. Two different types of angular velocity signals were examined: on the one hand, signals that are representative of a typical human motion analysis context; on the other, constant signals that allow to investigate the separate influence of angular velocity magnitude and 3D distribution. For what concerns the former, signals consistent with the angular velocity of pelvis and shank during level walking were chosen, as they are characterized by significantly different magnitudes and distributions. For what concerns the constant signals, several magnitudes and 3D distributions were considered in order to span the range of typical values encountered in human motion analysis.

## 2. Materials and Methods

The methodology presented in this study is based on the definition of a simulation framework developed using the MATLAB software (The MathWorks Inc., Natick, MA, USA). The simulator receives as input a reference 3D angular velocity signal and corrupts it with different types of disturbances (noise). The types of noise considered in this study are: residual bias (RB), scale factor (SF), white noise (WN), and bias instability (BI). The deterministic noise types (RB and SF) are represented by the uncompensated angular velocity offset and by the ratio between the measured and actual angular velocity, respectively. For what concerns the stochastic noise types, WN is described in terms of the standard deviation (STD) of the underlying process on each gyroscope axis, whereas BI is defined in terms of the bias instability coefficient as defined in [14]. For both stochastic noise types, five hundreds simulation runs were performed in order to characterize their mean effect and their variability.

The reference and the corrupted angular velocity signals were considered as those measured by an ideal and a noisy MEMS gyroscope, respectively. To obtain the orientations of these gyroscopes over time, the kinematic equation that relates the angular velocity with the time-derivative of the orientation was integrated, with an integration step within the typical range of MEMS gyroscope sampling frequencies. The integration of the kinematic equation was performed assuming that the angular velocity signals are constant within each interval of time between two subsequent samples [8]. To describe the orientation, the quaternion formalism was adopted as it presents several advantages with respect to other orientation parameterizations (*i.e*., orientation matrices and Euler angles), among which the lower computational load [17] and the smaller errors incurred in the process of numerical integration [18]. The relative orientation between the ideal and noisy gyroscopes was computed as the relative rotation that brings the noisy gyroscope onto the ideal one [19]. Finally, the orientation error was obtained as the root mean square (RMS) of the rotation angle associated to the relative orientation throughout the signal duration.

The above-described approach was used in this study with angular velocity signals of different nature: representative data of healthy gait and constant signals. Each of the considered types of noise was homogeneously distributed across the three gyroscope axes. In the cases in which the obtained results suggested that different noise distributions could be of interest, further tests were performed to assess the influence of noise directionality on the orientation error.

### 2.1. Effect of the Individual Types of Noise on the Orientation Error during Gait

**Figure 1:**
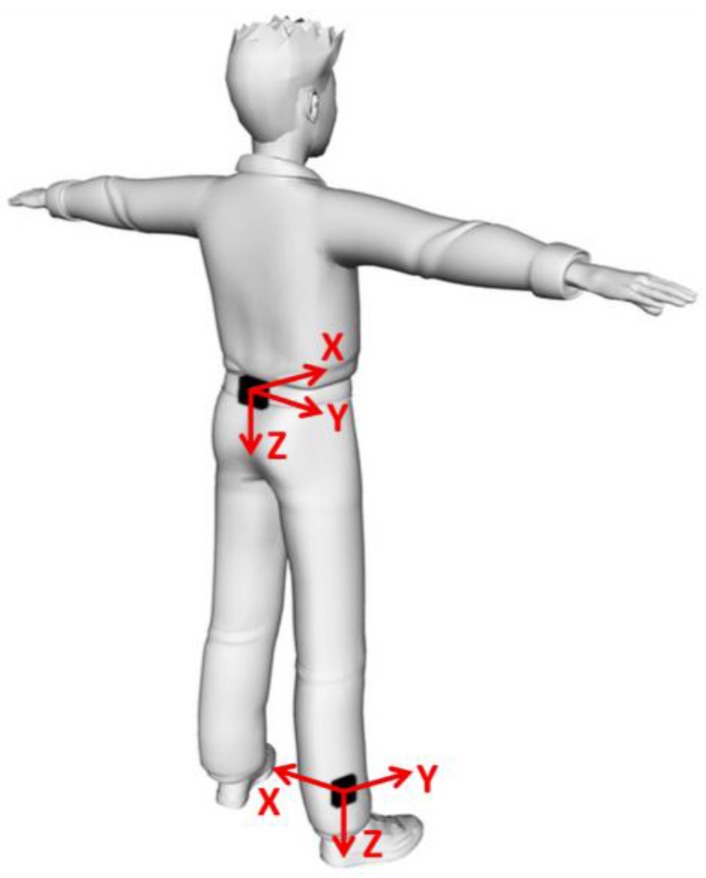
Sensor location and axes orientation of the two Magneto-Inertial Measurement Units (MIMUs) attached on the volunteer’s body (adapted from [9]).

To obtain reference signals representative of healthy gait, two synchronized MIMUs (Opal APDM Inc., Portland, OR, USA) embedding tri-axial MEMS gyroscopes were attached to the pelvis (L4–L5) and to the distal part of the right shank of a healthy male subject (31 years, 1.70 m, 68 kg) as shown in Figure 1. The subject was asked to walk along a 30 m straight pathway, for which twenty-three strides were recorded, with a sampling frequency of 128 samples/s. The participant gave his written informed consent according to the declaration of Helsinki. The research methodology described hereafter was approved by the institutional review board of the University of Rome “Foro Italico”.

Stride segmentation was performed by considering the angular velocity measured by the shank MIMU about its X axis, roughly corresponding to the medio-lateral direction [20,21]. Assuming the periodicity of gait, the angular velocity signals of both pelvis and shank were decomposed for each stride by using the Fourier series to obtain the frequency spectra [22]. The first 15 harmonics of the fundamental frequency (corresponding to the stride cycle) were considered [22]. The frequencies, amplitudes, and phases corresponding to each harmonic were averaged to produce the angular velocity signals of a typical stride of the performed gait. Reference signals of 60 s duration, considered as a typical value for the application of interest (*i.e*., human motion analysis), were thus generated for both pelvis and shank segments.

The reference signals were then corrupted with each individual noise type modelled within the simulation framework. In order to cover the range of typical values of commercially available MEMS gyroscopes, eleven levels (including the null noise value) were assigned to each noise type. The range of values was selected based on the literature [23] and on the technical specifications of off-the-shelf gyroscopes commonly employed in human movement analysis, like the ones used in this study. The eleven levels for WN and BI were equally distributed from the null noise value to a maximum value (0.05 rad/s and 0.002 rad/s, respectively). On the other hand, the levels of the deterministic noise types were chosen to represent positive and negative values, symmetrically distributed around the null noise value. For SF, the maximum noise level represents a 2.5% deviation from the ideal unit value. For what concerns RB, no reference value can be found, as the residual bias is not a characteristic of the gyroscope, but rather depends on the accuracy of the bias capture process. Since the bias capture consists in averaging a number N of noisy angular velocity samples, mostly affected by white noise (given the short time window considered in the bias capture), the uncertainty of the estimated bias in terms of STD can be approximated by scaling the STD of the white noise by $\sqrt{\text{N}}$. Hence, considering the maximum WN level (0.05 rad/s) and a bias capture of about N = 500 samples [9], the variability of the process is 0.0022 rad/s on each gyroscope axis. This value was chosen as maximum residual bias on each axis, which therefore corresponds to a maximum noise level associated to the magnitude of the RB vector of 0.004 rad/s.

The orientation error previously defined was evaluated for each level of each noise type and for both pelvis and shank locations. For the stochastic noise types (WN and BI), the testing for differences between the pelvis and shank locations was carried out using inferential statistics. First, the normality of the distribution of the orientation errors was checked with the Shapiro-Wilk test. In case of skewed distribution, differences between the two locations were tested through a Mann-Whitney *U* as a non-parametric method. Otherwise, in case of normal distribution, the differences were tested through an unpaired *t*-test (α = 0.05). For the deterministic noise types (SF and RB) the differences between the two locations were quantified through the RMS difference. To investigate the trend with which the orientation error due to each noise type increases with the noise levels, polynomial curve fittings were performed (for WN and BI both on their means and STDs) and their adequacy described in terms of coefficient of determination (*R*^2^). The statistical analysis was performed using IBM SPSS Statistics (IBM Corp., Armonk, NY, USA).

### 2.2. Influence of the Angular Velocity 3D Distribution and Magnitude on the Orientation Error for Each Noise Type

The procedures implemented to single out the effect of the angular velocity magnitude and 3D distribution on the orientation error due to each individual noise type are described in the following sections.

#### 2.2.1. Angular Velocity 3D Distribution

To assess the effect of the 3D distribution of the angular velocity ω across the three gyroscope axes, the magnitude of the angular velocity was set to 1 rad/s. The locus of the possible 3D distributions of this unit vector is represented by the surface of a unit sphere in the [$\omega_{x}$ $\omega_{y}$ $\omega_{z}$] space. The surface was then discretized considering intervals of 10° for the polar angle α and intervals of the azimuth angle ranging from 10° (when α = 90°) to 360° (when α = 0° and α = 180°), leading to about 900 combinations of $\omega_{x}$ $\omega_{y}$ $\omega_{z}$ components, describing points on the sphere’s surface. Constant signals of 60 s duration, sampled at the same frequency as the MEMS gyroscope described above (Section 2.1), were generated for each of these combinations, and these reference signals were then corrupted with each of the four above-mentioned noise types, using the maximum value of the levels previously described (Section 2.1). Five hundred simulations were run for the two stochastic noise types (WN and BI). The distribution of the orientation error, associated to each combination of $\omega_{x}$ $\omega_{y}$ $\omega_{z}$ components across the sphere, was then obtained for each noise type.

#### 2.2.2. Angular Velocity Magnitude

To assess the individual contribution of the angular velocity magnitude to the orientation error due to each noise type, the magnitude of the angular velocity was varied to cover the range of typical values observed in gait at pelvis and shank levels (from 0 to 10 rad/s). For each magnitude value, the angular velocity was studied for the 3D distributions that were found to be most paradigmatic (e.g., corresponding to conditions of maximum or minimum errors) according to the results of the previous section (Section 2.2.1). Constant signals of 60 s duration were generated for each magnitude and distribution, sampled at the same frequency as the MEMS gyroscope used for the experimental acquisitions (Section 2.1). These reference signals were then corrupted with each of the four noise types, using the maximum value of the levels previously described (Section 2.1), and the corresponding orientation errors were evaluated. For the stochastic noise types (WN and BI), the normal distribution of the orientation errors was checked with the Shapiro-Wilk test and the differences between the orientation errors associated to the identified paradigmatic 3D distributions and to the different angular velocity magnitudes were assessed through parametric or non-parametric tests according to the results of the normality test. Specifically, when testing for angular velocity distribution differences, an unpaired *t*-test or a Mann-Whitney *U* test was performed, according to the normality test. On the other hand, when testing for differences among angular velocity magnitudes, a one-way ANOVA or a Kruskal-Wallis *H* test was carried out, since more than two datasets were compared. If a significant magnitude effect was found, *post-hoc* comparisons were performed between each pair of angular velocity magnitudes using either the Bonferroni correction or a Mann-Whitney *U* test. For what concerns the deterministic noise types (SF and RB), the differences in orientation error were assessed through the RMS difference.

## 3. Results

**Figure 2:**
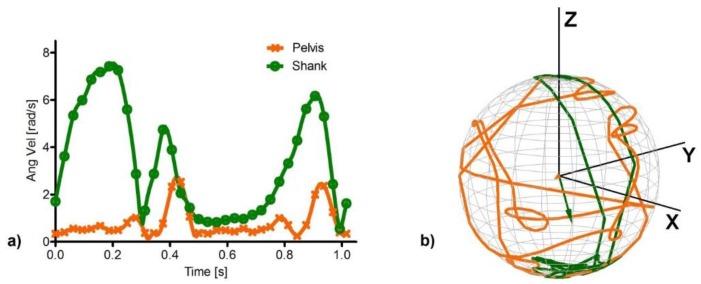
Magnitude (**a**) and direction (**b**) of the angular velocity vector associated to the pelvis (orange) and shank (green) gyroscopes during a stride; The orange and green arrows (**b**) represent the position of the centroids for the trajectory followed by the unit vector describing the direction of the angular velocity of pelvis and shank, respectively.

The differences in the angular velocity observed at the pelvis and shank during a stride are shown in Figure 2, both in terms of magnitude and 3D distribution. For what concerns the magnitude (Figure 2a), the values at the pelvis are lower both in mean and peak-to-peak values than at the shank. Mean values for the pelvis and the shank angular velocity magnitude were, in fact, 0.53 rad/s and 1.76 rad/s, respectively. Regarding the 3D distribution (Figure 2b), the trajectory followed by the unit vector of the angular velocity in the pelvis and shank is reported, as well as the centroids of the two trajectories: their direction represents the mean direction of the angular velocity and their norm reflects how dispersed the angular velocity distribution is during a stride [24]. The smaller is the norm of the centroid locations, the more uniform is the corresponding distribution, and the larger the norm, the stronger is the directionality of the angular velocity. Therefore, the pelvis presents a more variable 3D distribution than the shank, which in turn is characterized by a clearer directionality.

### 3.1. Homogeneously Distributed Noises

The effect of homogeneously distributed individual noise types on the orientation error and the influence of the angular velocity 3D distribution and magnitude are presented in the following sections.

#### 3.1.1. Effect of the Individual Types of Noise on the Orientation Error during Gait

**Figure 3:**
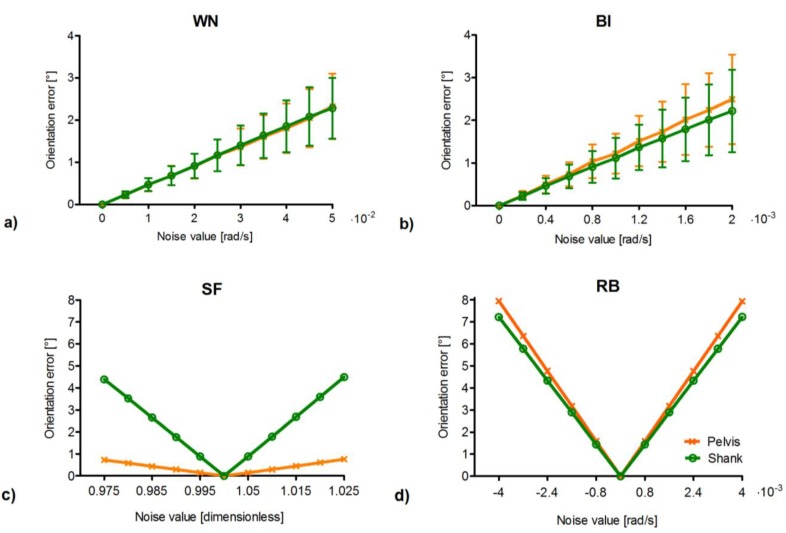
Orientation error associated to each homogeneously distributed noise type as a function of noise levels for the ideal pelvis (orange) and shank signals (green). (**a**) WN: white noise; (**b**) BI: bias instability; (**c**) SF: scale factor; and (**d**) RB: residual bias. For WN and BI (**a**,**b**) the abscissae indicate the noise level values associated to each gyroscope axis, and the orientation error is reported as mean and standard deviation (vertical bars) corresponding to the five hundred simulation runs of each noise level. For SF and RB (**c**,**d**) the abscissae indicate the magnitude of the noise level values. Noise levels are expressed in the International System of Units, whereas the orientation error is expressed in degrees (°) to facilitate its interpretation. (Please note that, for WN, BI, and RB, multiplication factors of 10^−2^ or 10^−3^ are reported on the right of the abscissae).

The orientation error due to different levels of homogeneously distributed individual noise types, affecting the angular velocity signals representative of the motion of pelvis and shank during gait, is shown in Figure 3.

For what concerns the stochastic noise types, the mean orientation error due to WN and its standard deviation were found to increase with the values of the noise levels following a linear trend (*R*^2^ = 0.99), in agreement with [12]. Moreover, no significant difference was encountered between the errors corresponding to the pelvis and shank for each noise level. Regarding BI, the orientation error increased proportionally to the values of the noise levels (*R*^2^ = 0.99), similarly to WN. However, in this case, significant differences between the pelvis and the shank locations were found (t_(998)_ = 2.81, *p* < 0.01), with greater errors associated to the pelvis with respect to the shank.

As for the deterministic noise types, linear trends were observed for the orientation errors due to SF (*R*^2^ = 0.99), the slope of which was found to be different for the pelvis and shank locations (RMS differences = 2.4°). In fact, since the shank experiences mean angular velocities (µ = 1.76 rad/s) that are greater than those of the pelvis (µ = 0.53 rad/s) (Figure 2a), the former body segment is more sensitive to the SF noise type. Finally, for what concerns RB, the orientation error was found to increase with the values of the noise levels following a linear trend (*R*^2^ = 0.99), in accordance with [12]. The effects of the non-linear relationship between angular velocity and orientation [8] are revealed by the fact that the two sensors present different slopes (RMS differences = 0.5°), indicating that the same bias applied to different angular velocity curves yields different variations in orientation. Similarly to BI, the orientation error associated to the pelvis was greater than that at the shank.

#### 3.1.2. Influence of the Angular Velocity 3D Distribution and Magnitude on the Orientation Error for Each Noise Type

**Figure 4:**
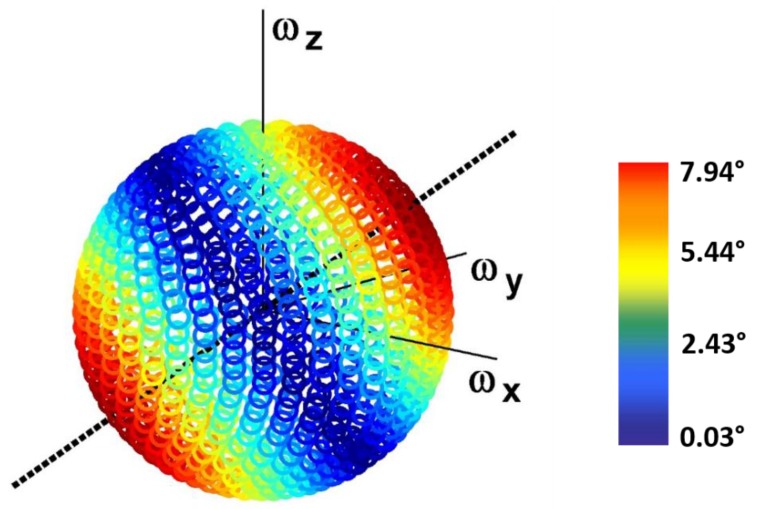
Distribution of the orientation error due to the residual bias RB with equally distributed components across the three axes (directed as the dashed black line in the figure) across the sphere representing different 3D distributions of a constant unit angular velocity vector. The error is represented by a color range, from dark red (maximum values) to dark blue (minimum values).

For what concerns the effect of the angular velocity 3D distribution, assessed considering different directions of a constant unit angular velocity vector, it was found to affect the orientation error due to homogeneously distributed noise types only in the case of RB. The distribution of the orientation error due to RB across the sphere is shown in Figure 4. Maximum errors (shown in red) correspond to the conditions in which the components of the angular velocity are equal, in magnitude and sign, across the three axes, and match the intersection of the sphere with the black axis, which represents the direction of the considered RB vector.

**Figure 5:**
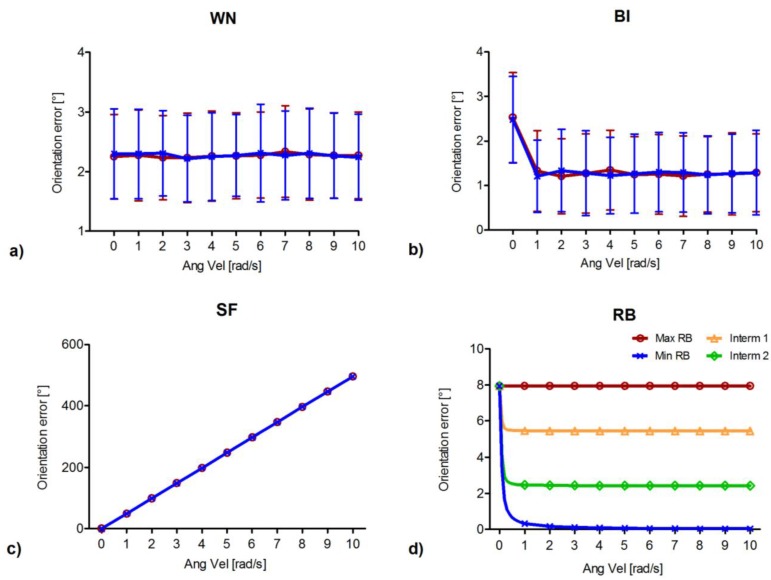
Orientation error associated to each homogeneously distributed noise type as a function of the magnitude of a constant angular velocity vector. (**a**) WN: white noise; (**b**) BI: bias instability; (**c**) SF: scale factor; and (**d**) RB: residual bias. Four distributions of the angular velocity vectors (maximum RB error: dark red; minimum RB error: dark blue; intermediate RB errors: yellow and green) were considered. Results for WN, BI and SF only report the directions of maximum and minimum RB error. The orientation error for WN and BI is reported as mean and standard deviation (vertical bars) corresponding to the five hundred simulation runs of each noise level.

The effect of the angular velocity magnitude on the orientation error due to homogeneously distributed noise types is reported in Figure 5. As the distribution of the angular velocity across the three axes was found to affect the orientation error (due to RB, Figure 4), the most paradigmatic distributions were considered. To represent the conditions of maximum error (dark red poles on the sphere, Figure 4) the direction corresponding to positive and equally distributed angular velocity components across the three axes was selected; to represent the conditions of minimum error (dark blue equator on the sphere), a point on the circumference perpendicular to the direction of equally distributed angular velocity components and with maximum radius was selected; finally, two intermediate directions were considered, by selecting two points on circumferences perpendicular to the direction of equally distributed angular velocity components (green and yellow parallels on the sphere). As the orientation errors due to WN, BI and SF were found not to be affected by the distribution of the angular velocity across the three axes (Section 3.1.2), for sake of clarity only the results corresponding to the two extreme distributions are reported in Figure 5.

For what concerns the stochastic noise types, no significant difference was encountered among the orientation errors corresponding to the different angular velocity magnitudes for WN (χ^2^_(10)_ = 6.93, *p* = 0.73). On the other hand, a significant magnitude effect was observed for BI (χ^2^_(10)_ = 619.51, *p* < 0.001). A Mann-Whitney *U* test, performed between each pair of angular velocity magnitudes, confirmed the presence of significant differences between the first angular velocity magnitude (null value) and each remaining magnitudes (Mann-Whitney *U* = 45058, *p* < 0.001), with larger errors associated to low magnitudes. This result matches with the above mentioned larger orientation error displayed by the pelvis with respect to the shank, when BI was considered (Figure 3b). For both WN and BI, no significant difference was found between the orientation errors corresponding to the two extreme distributions considered (red and blue curves in Figure 5a,b), the lowest *p*-value being 0.1, (Mann-Whitney *U* = 117,036). This result supports the fact that, for these noise types, the 3D distribution of the angular velocity vector does not affect the orientation error.

Regarding SF, the orientation errors was found to linearly depend on the magnitude of the angular velocity, in agreement with [12], whereas the RMS difference between the two extreme distributions was found to be 2 × 10^−8^ degrees, indicating that the 3D distribution of the angular velocity did not to affect the orientation estimation accuracy.

Finally, for what concerns RB, the effect of the angular velocity magnitude on the orientation error was found to vary according to the angular velocity 3D distribution (RMS difference between the two extreme distributions = 7.25°), except in the case limit of null magnitude, where the orientation error corresponds to the integration of the sole residual bias and its value is constant (Figure 5d, Max RB). In all other cases, the orientation error was found to vary from the maximum values (for ||ω||=0) to a horizontal asymptote. The greater orientation error at low angular velocity magnitudes and the horizontal asymptote at higher magnitudes can also be observed for BI (Figure 5b). This common behavior is to be attributed to the fact that both types of noise represent a bias of the angular velocity. Nevertheless, it should be noticed that the orientation error due to BI is not affected by the angular velocity 3D distribution, as the stochastic nature of BI produces randomly oriented biases and therefore a null mean effect.

### 3.2. Other Paradigmatic Noise Distributions

If only homogeneous distributions of noise types are considered, it is unclear whether the maximum errors associated to RB depend on the direction of the angular velocity vector or rather on the relative orientation of the angular velocity vector with respect to the RB vector. For this reason, an RB vector with non-equally distributed components across the three axes was selected, with which the three tests considered in this study (Section 2.2, Section 2.2.1 and Section 2.2.2) were repeated. Among the possible distributions of the RB components, we chose to consider RB aligned to a relevant direction encountered in the gait scenario considered in this study: the mean direction of the angular velocity vector measured by the shank gyroscope (Figure 2b). With respect to the case of equally distributed RB components, the same noise values (in terms of RB magnitude) were considered, only distributed differently across the three axes.

#### 3.2.1. Effect of the RB Noise on the Orientation Error during Gait

**Figure 6:**
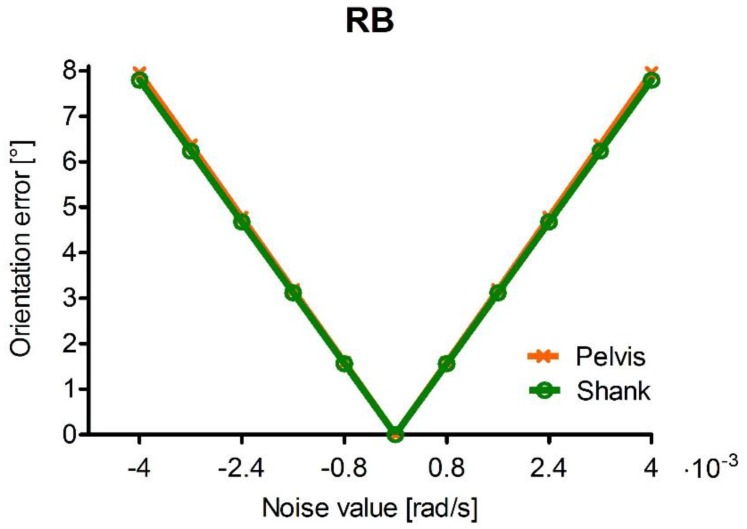
Orientation error associated to an RB aligned with the mean direction of the angular velocity in the shank as a function of noise levels for the ideal pelvis (orange) and shank signals (green). The abscissa indicates the magnitude of the noise level values. (Please note that a multiplication factor of 10^−3^ is reported on the right of the abscissa).

The effect of different levels of RB noise on the orientation error, considering the distribution defined above, was evaluated for the angular velocity signals representative of the motion of pelvis and shank during gait (Figure 6). With respect to the case of equally distributed RB components, the pelvis is hardly affected by the different noise distribution (the greatest change in error is about 0.1%), whereas the error associated to the shank increases and tends to match the error at the pelvis.

#### 3.2.2. Influence of the Angular Velocity 3D Distribution and Magnitude on the Orientation Error for the RB Noise

**Figure 7:**
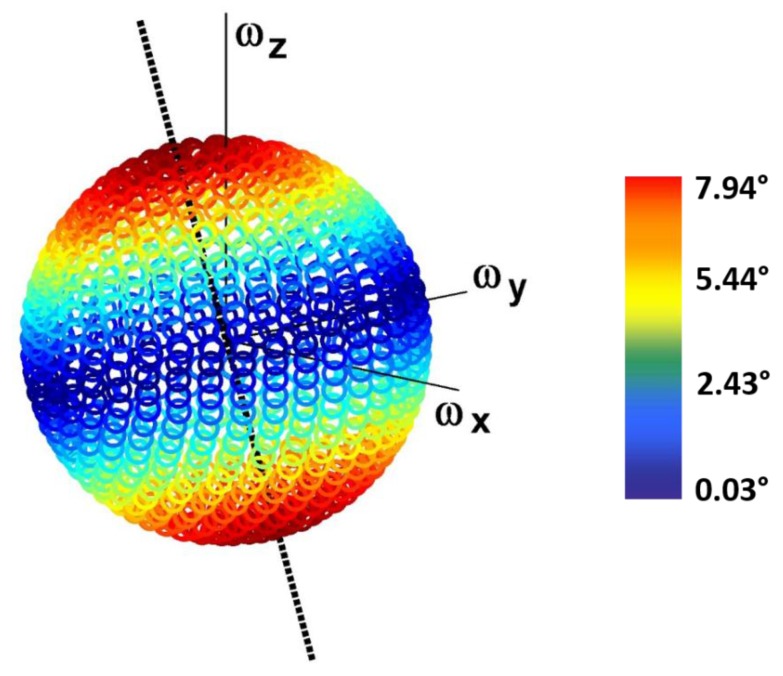
Distribution of the orientation error due to an RB aligned with the mean direction of the angular velocity in the shank across the sphere representing different 3D distributions of a constant unit angular velocity vector. The error is represented by a color range, from dark red (maximum values) to dark blue (minimum values). The dashed black line represents the direction of the RB vector.

The distribution of the orientation error due to the maximum noise level of the considered non-equally distributed RB components is shown in Figure 7. It should be noted that the maximum errors (shown in red) correspond to the conditions of alignment between the angular velocity vector and the direction of the RB vector (black line), whereas minimum errors (shown in blue) occur when the two vectors are orthogonal.

Finally, the effect of the angular velocity magnitude on the orientation error due to the maximum level of the non-equally distributed RB components was evaluated. The paradigmatic directions were defined following the same criterion as for the equally distributed RB case, in order to represent the conditions of maximum error, minimum error, and the two intermediate conditions. For this, a transformation was applied to the paradigmatic directions identified on the sphere in Figure 4, in order to select the same conditions on the sphere in Figure 7 with respect to the RB direction. The errors corresponding to each paradigmatic direction of the angular velocity were found to match those obtained when RB is distributed equally across the three axes (Figure 5d), with an average difference in the order of 10^−8^ degrees.

## 4. Discussion

The present study focuses on the effect of the most relevant stochastic and deterministic noise types typical of MEMS gyroscopes on the accuracy of the orientation estimated through numerical integration in the context of human movement analysis. Reference signals representative of the angular velocity of pelvis and shank during gait were considered, and corrupted with each of the four considered noise types: white noise, bias instability, scale factor and residual bias. As the signals differ significantly in the two locations in terms of both magnitude and 3D distribution across the gyroscope axes, the influence of each of these two parameters was assessed, in order to gain further insight into the effect of each noise type on the accuracy of the estimated orientation.

For what concerns WN, both angular velocity magnitude and 3D distribution were found not to affect the orientation error, indicating that this error is determined exclusively by the white noise STD, regardless of the angular velocity signal it is applied to.

Regarding BI and SF, not only the orientation error increased with the noise level, similarly to WN, but also it was found to be influenced by the angular velocity magnitude. In this respect, a different trend was observed for the two noise types: while larger orientation errors were associated to low angular velocity magnitudes for BI, the opposite behavior was obtained for SF, in agreement with [12]. On the other hand, the 3D distribution of the angular velocity was found not to affect the orientation estimation accuracy for both noise types.

Interesting results were obtained for RB, for which the orientation error was found to be strongly influenced by both angular velocity magnitude and 3D distribution, as it was found to vary up to 8° in the tested conditions. Specifically, the results obtained for an RB vector with equally and non-equally distributed components (Figure 4 and Figure 7, respectively) indicate that the maximum orientation error occurs when the angular velocity and RB vectors are aligned, whereas orthogonality between the two vectors leads to a minimum error. Moreover, for both equally and non-equally distributed RB components, the four paradigmatic directions of the angular velocity (defined in order to represent the same alignment/misalignment conditions with respect to the RB vector) were found to yield the same orientation errors (Figure 5d, Section 3.2.2). The fact that what is decisive is the direction of the RB vector with respect to the angular velocity vector is further supported by the analytical relationship between the orientation error and RB, reported in the Appendix. As for the effect of the angular velocity magnitude ||ω||, it was found to depend on the relative orientation between the angular velocity and the RB vectors. When the angular velocity and RB vectors were not aligned, the orientation error was found to vary from the maximum values (for ||ω||=0) to a horizontal asymptote. Both the value of the asymptote and the rate with which it is approximated depend on the relative orientation between the angular velocity and the RB vectors: the greater the misalignment, the slower was the tendency towards the asymptote and the smaller was the error, which reached remarkably small values when the angular velocity was orthogonal to RB (asymptote of the blue curve). It is worth to note that the effect of the magnitude on the orientation accuracy is mostly evident when its values are relatively small (approximately ||ω||<1 rad/s), whereas, for greater magnitudes, the orientation error is determined almost entirely by the 3D distribution. Hence, it should be noted that the same angular velocity, and the same value of RB, can lead to a great variability in orientation error simply due to the relative orientation between the two vectors as seen by the gyroscope axes.

These results expose the inadequacy of the signal-to-noise ratio as a figure of merit of the angular velocity signal when it is employed for orientation estimation. Additionally, they point out the substantial contribution of the spatial orientation of the gyroscope, determined by its positioning, to the orientation error due to RB. In fact, given an angular velocity vector, which is determined by the movement under analysis, the condition of alignment or misalignment between the measured angular velocity and the RB vectors is determined by the spatial orientation of the gyroscope. However, it must be noticed that in real experimental conditions, the direction of RB is unknown, and therefore it is hardly possible to provide practical guidelines to establish the conditions of minimum error in terms of optimal gyroscope positioning.

For what concerns the alignment/misalignment of the pelvis and shank angular velocity vectors with RB, it may be noted that the error due to RB vectors with equally and non-equally distributed components (Figure 3d and Figure 6, respectively) is basically unchanged in the pelvis, whereas for the shank it is lower when RB is equally distributed across the axes and tends to match the error in the pelvis when RB is aligned with the mean direction of the angular velocity in the shank itself. The reason behind these two different behaviors lies in the different uniformity characteristics of the distribution of the angular velocity direction during gait in the two considered locations. In fact, the angular velocity of the shank presents a much stronger directionality with respect to the pelvis, which in turn shows a much more uniform 3D distribution during a stride (Figure 2). Therefore, the direction of RB does not substantially affect the value of the corresponding orientation error in the pelvis, as any instantaneous condition of alignment/misalignment between the angular velocity and RB vectors is incidental. On the other hand, as the angular velocity in the shank presents a clearer directionality, the direction of the RB vector affects the error more significantly. By choosing RB to match the mean direction of the angular velocity, the two vectors were more closely aligned during most of the stride, increasing the orientation error with respect to the case in which the RB components were distributed equally across the three axes.

It should be noted that, due to the varying magnitude and direction of the pelvis and shank angular velocities during gait, the effect of different RB component distributions on the orientation error is slighter than when constant angular velocities are considered. Nevertheless the considerations presented in this study are still valid in principle as integration methods generally consider the angular velocity constant in each time interval between two subsequent samples [25]. Additionally, it should be noted that the effect of the signal sampling frequency on the accuracy of the integration process and its influence on the orientation error due to the considered noise types has not been investigated in this study and may deserve further consideration. Another aspect that has not been included in the present work and that may be of interest is the error due to gyroscope axes misalignment: further studies should be carried out to assess the influence of this feature on orientation estimation accuracy. Finally, the results of the present study should be extended to real-life scenarios with caution, as in reality all types of noise are present at once and their effects are combined in unpredictable ways. Nevertheless, the simulation approach followed in this work was needed to reveal the effect of each gyroscope noise type on the orientation error, contributing to the understanding of the phenomena and the main issues involved in the orientation estimation through gyroscope signal integration. It is worth noting that, in several real-life applications, sensor fusion techniques can be employed to reduce some of the errors discussed above [8]. The extent to which this reduction can be achieved strongly depends on the sensor fusion algorithm employed and on the disturbances characterizing the accelerometer and magnetometer sensors [9,26], and its assessment is beyond the scope of this paper.

## 5. Conclusions

The present study is based on a simulation approach and focuses on the individual effect of four types of noise, typical of MEMS gyroscopes, on the accuracy of the orientation estimation through numerical integration, applied to a human movement context. Additionally, the influence of the angular velocity magnitude and 3D distribution on the orientation estimation accuracy was assessed considering appropriate constant signals.

All noise types were found to affect the orientation error that increases with the noise levels. The magnitude of the angular velocity was found to affect the orientation error due to all noise types except for the white noise. The error due to the scale factor increases proportionally with the angular velocity magnitude, whereas, for what concerns both the bias instability and the residual bias, the error decreases with a horizontal asymptote when the angular velocity magnitude increases. Only the error due to the residual bias was found to depend on the distribution of the angular velocity about the three gyroscope axes. Specifically, the orientation error depends on the mutual alignment of the angular velocity and residual bias vectors: maximum errors occur when the two vectors are aligned and minimum errors correspond to a condition of orthogonality between the two. The same angular velocity and residual bias were found to yield an orientation error that varies significantly (up to 8° in the tested conditions) depending on their mutual alignment.

These results lead to two main conclusions. First, the angular velocity signal-to-noise ratio alone cannot be used to represent the accuracy with which the orientation can be estimated. Second, special attention must be paid in providing and interpreting measures of accuracy for orientation estimation algorithms, as orientation accuracy measures have been proven to be meaningless unless appropriately contextualized. Specifically, static accuracy information can be provided by a single value, as is currently done in gyroscope datasheets, whereas dynamic accuracy requires to specify the conditions in which it was assessed, as it is affected by the very angular velocity signal being measured. Standardized conditions could be defined, under which producers should carry out the dynamic accuracy assessment, in order to allow users to compare gyroscope performances on equal terms.

## Appendix

In this appendix the relationship between a constant reference angular velocity signal corrupted with a residual bias (RB) and the consequent orientation error is derived.

Let $\omega_{i}$ and $\omega_{n}$ denote ideal and noisy angular velocity 3D signals, respectively, related through the expression $\omega_{n}=\omega_{i}+b$, with b indicating the RB vector.

Assuming an initial condition $q_{0}={\left[\begin{array}{cc} \begin{array}{cc} 0 & 0 \end{array} & \begin{array}{cc} 0 & 1 \end{array} \end{array}\right]}^{T}$, the quaternions, propagated according to the ideal and noisy angular velocities, are respectively:

(A1) $$q_{i}=[\begin{array}{c} \mathrm{sinc}\left(\frac{||\omega_{i}||}{2\;f_{s}}\right)\frac{\omega_{i}}{2\;f_{s}}\\ \cos\left(\frac{||\omega_{i}||}{2\;f_{s}}\right) \end{array}];\;q_{n}=[\begin{array}{c} \mathrm{sinc}\left(\frac{||\omega_{n}||}{2\;f_{s}}\right)\frac{\omega_{n}}{2\;f_{s}}\\ \cos\left(\frac{||\omega_{n}||}{2\;f_{s}}\right) \end{array}]$$

where $f_{s}$ represents the sampling frequency.

For a generic integration step, within which the angular velocity is considered constant [8], the scalar part of the error quaternion $∆q_{s}$ is given by [27]:

(A2) $$∆q_{s}=\cos\left(\frac{||\omega_{i}||}{2\;f_{s}}\right)\cos\left(\frac{||\omega_{n}||}{2\;f_{s}}\right)+\frac{1}{4\;f_{s}^{2}}\text{sinc}\left(\frac{||\omega_{i}||}{2\;f_{s}}\right)\text{sinc}\left(\frac{||\omega_{n}||}{2\;f_{s}}\right)\left(\omega_{i}\cdot \omega_{n}\right)$$

Given the relationship between $\omega_{i}$ and $\omega_{n}$, Equation (A2) can be rewritten as:

(A3) $$∆q_{s}=\cos\left(\frac{||\omega_{i}||}{2\;f_{s}}\right)\cos\left(\frac{||\omega_{n}||}{2\;f_{s}}\right)+\frac{1}{4\;f_{s}^{2}}\text{sinc}\left(\frac{||\omega_{i}||}{2\;f_{s}}\right)\text{sinc}\left(\frac{||\omega_{n}||}{2\;f_{s}}\right){||\omega_{i}||}^{2}+\;+\frac{1}{4\;f_{s}^{2}}\text{sinc}\left(\frac{||\omega_{i}||}{2\;f_{s}}\right)\text{sinc}\left(\frac{||\omega_{n}||}{2\;f_{s}}\right)\left(\omega_{i}\cdot b\right)$$

The relationship between the error angle ∆θ and $∆q_{s}$ is [27]:

(A4) $$∆\theta =2\text{acos}\left(∆q_{s}\right)$$

Assuming low bias values ($b\ll \omega_{i}$), the following conditions hold:

- On the one hand, the noisy and ideal angular velocities tend to match:
  (A5) $$\omega_{n}\approx \omega_{i}$$

Therefore, Equation (A3) can be simplified to:

(A6) $$∆q_{s}\approx k\left(\omega_{i},f_{s}\right)+\frac{1}{4\;f_{s}^{2}}{\text{sinc}}^{2}\left(\frac{||\omega_{i}||}{2\;f_{s}}\right)||\omega_{i}||||b||\cos\beta$$

where β is the angle between the angular velocity and the RB vectors.

- On the other hand, the scalar part of the error quaternion tends to unity:
  (A7) $$∆q_{s}\;\approx 1$$

Therefore, with a first-order Taylor approximation in the neighborhood of 1, Equation (A4) becomes:

(A8) $$∆\theta \approx 2\text{acos}\left(∆q_{s}\right)\approx -2\;∆q_{s}$$

Finally, substituting Equation (A.6) into Equation (A8), the following Equation is obtained:

(A9) $$∆\theta \approx -2\;∆q_{s}\approx k_{1}\left(\omega_{i},f_{s}\right)+\frac{1}{2\;f_{s}^{2}}{\text{ sinc}}^{2}\left(\frac{||\omega_{i}||}{2\;f_{s}}\right)||\omega_{i}||||b||\cos\beta$$

where $k\left(\omega_{i},f_{s}\right)$ and $k_{1}\left(\omega_{i},f_{s}\right)$ are functions of $\omega_{i}$ and $f_{s}$.

According to Equation (A9), the absolute value of the rotation ∆θ depends on the relative orientation between these two vectors, and therefore, so does its RMS, assumed as error metric in this paper. In particular, given the angular velocity and RB magnitudes, ||∆θ|| is maximum when β=kπ, *i.e*., the two vectors are aligned (or counter-aligned), and it is minimum when $\beta =\frac{\pi }{2}+k\pi$, *i.e*., the two vectors are orthogonal.
